# Cavity Shave Margins in Breast Conservative Surgery a Strategy to Reduce Positive Margins and Surgical Time

**DOI:** 10.3390/curroncol31010035

**Published:** 2024-01-16

**Authors:** Gianluca Vanni, Marco Pellicciaro, Giulia Renelli, Marco Materazzo, Amir Sadri, Valentina Enrica Marsella, Federico Tacconi, Sebastiano Angelo Bastone, Benedetto Longo, Giordana Di Mauro, Valerio Cervelli, Massimiliano Berretta, Oreste Claudio Buonomo

**Affiliations:** 1 Breast Unit Policlinico Tor Vergata, Department of Surgical Science, Tor Vergata University, Viale Oxford 81, 00133 Rome, Italy; vanni_gianluca@yahoo.it (G.V.); marco.pellicciaro@alumni.uniroma2.eu (M.P.); giulia.renelli@alumni.uniroma2.it (G.R.); mrcmaterazzo@gmail.it (M.M.); valentinaenrica.marsella@ptvonline.it (V.E.M.); benedettolongo@gmail.com (B.L.); oreste.buonomo@ptvonline.it (O.C.B.); 2 PhD Program in Applied Medical-Surgical Sciences, Department of Surgical Science, Tor Vergata University, 00133 Rome, Italy; sebastianoangelo.bastone@ptvonline.it; 3 Plastic Surgery, Great Ormond Hospital for Children NHS Foundation Trust, London WC1N3JH, UK; info@amirsadri.com; 4 Unit of Thoracic Surgery, Department of Surgical Sciences, Tor Vergata University, 00133 Rome, Italy; tacconifederico@gmail.com; 5 Plastic and Reconstructive Surgery at Department of Surgical Science, Tor Vergata University, 00133 Rome, Italy; valeriocervelli@virgilio.it; 6 Medical Oncology Unit, Department of Human Pathology “G. Barresi”, University of Messina, 98122 Messina, Italy; giordana.di.mauro@hotmail.com; 7 Department of Clinical and Experimental Medicine, University of Messina, 98122 Messina, Italy

**Keywords:** breast cancer, cavity shave, positive margins, reduce re-excision, reducing surgical time

## Abstract

**Background:** Resection of additional tissue circumferentially around the cavity left by lumpectomy (cavity shave) was suggested to reduce rates of positive margins and re-excision. **Methods:** A single center retrospective study which analyzed margins status, re-excision, and surgical time in patients who underwent breast conserving surgery and cavity shave or intraoperative evaluation of resection margins. **Results:** Between 2021 and 2023, 594 patients were enrolled in the study. In patients subjected to cavity shave, a significant reduction in positive, focally positive, or closer margins was reported 8.9% vs. 18.5% (*p* = 0.003). No difference was reported in terms of surgical re-excision (*p* < 0.846) (5% vs. 5.5%). Surgical time was lower in patients subjected to cavity shave (<0.001). The multivariate analysis intraoperative evaluation of sentinel lymph node OR 1.816 and cavity shave OR 2.909 were predictive factors for a shorter surgical time. Excluding patients subjected to intraoperative evaluation of sentinel lymph node and patients with ductal carcinoma in situ, patients that underwent the cavity shave presented a reduced surgical time (67.9 + 3.8 min vs. 81.6 + 2.8 min) (*p* = 0.006). **Conclusions:** Cavity shaving after lumpectomy reduced the rate of positive margins and it was associated with a significant reduction in surgical time compared to intraoperative evaluation of resection margins.

## 1. Introduction

Breast cancer is the most common neoplasia in women in terms of incidence and the second in terms of mortality [1]. Both survival and incidence are characterized by an increasing trend, attributed to the increased accessibility to screening programs leading to more frequent early diagnoses and new systemic therapies [1]. This results in improved outcomes and the possibility of opting for conservative surgery rather than mastectomy and, consequently, better quality of life for patients [2].

Breast conserving surgery is defined as a surgical excision of the primary tumor and a small amount of surrounding disease-free tissue, with maximal tissue preservation, and achieving negative margins [2].

The current guidelines from ASCO (American Society of Clinical Oncology), SSO (Society of Surgical Oncology), ASTRO (American Society for Radiation Oncology), and NCCN (National Comprehensive Cancer Network) recommend adopting the “no ink on the tumor” as the definition of a negative margin for invasive breast cancer undergoing lumpectomy [2,3].

Conversely, for ductal carcinoma in situ, the guidelines recommend margins of at least 2 mm [2,3,4].

Positive margins are associated with an increased incidence of local recurrence [5,6]. The majority of patients with positive or focally positive margins are subjected to re-excision with a second surgical procedure. This has a negative psychological affect and quality of life [6].

Preserving as much healthy glandular tissue as possible to enhance aesthetic outcomes, while concurrently achieving negative margins for oncological reasons remains the central and challenging objective of oncoplastic surgery [7]. Avoidance of tissue removal during lumpectomies in order to spare the glandular tissue leads to a drastic reduction in free margin width between healthy tissue and the tumor, resulting in a higher number of positive margins and consequent need for surgical re-excision [7].

For decades, intra-operative histological frozen section evaluation of the resection margins during breast conserving surgery has been the gold standard [6]. However, this technique has been criticized in the literature both for the rate of resulting ‘false positives’ and a trend towards increased operative and anesthesia times [8].

The cavity shave technique involves minimal resection of the breast lesion and the extension of all margins to complete a total clearance of the residual breast tissue [7]. According to data reported in the literature, cavity shaving seems to reduce the rate of positive margins and re-excisions, whilst ensuring oncological safety without compromising aesthetic outcomes [7,9]. Despite these advantages, the technique adopted for intra-operative evaluation of resection margin in breast conserving surgery can vary depending on tumor and surgeon factors [10,11].

The aim of this retrospective study was to evaluate the potential benefits of cavity shave for the management of resection margin in breast conserving surgery.

## 2. Materials and Methods

All patients with a diagnosis of breast cancer who underwent breast conserving surgery from September 2021 to September 2023 at the Breast Unit of the U.O.S.D. at Policlinico Tor Vergata were evaluated in this study. The retrospective manuscript was approved by the local Ethical Committee (Approval number 72/23).

All data were retrieved from clinical notes and pathological reports.

Pre-operative diagnosis was confirmed by fine needle aspiration, micro-biopsy, vacuum-assisted biopsy, or vacuum-assisted excision. Pre-operative breast cancer histologic subtype, invasive or in situ lesions, and prognostic and predictive factors were collected for analysis.

Breast conservative surgeries included all the procedures with partial gland removal. All procedures were performed by an oncoplastic surgeon. When possible, lumpectomy was the main procedure performed with oncoplastic principles. Oncoplastic surgical techniques usually involve a large dissection, and they are divided into two sub-types. Oncoplastic level I consists of reshaping of the residual gland and it is based on volume displacement, rearrangement, and aesthetic scar placement [12]. Oncoplastic level II is considered when complex specific oncoplastic techniques are adopted based on volume reduction with breast lift (mastopexy) and symmetrization, or volume replacement with nearby tissue flap or fat grafting [12]. Due to larger resection volumes in oncoplastic level II, this cohort was excluded from the study. Patients subjected to mastectomy were also excluded.

Concomitant surgical procedure for lymph nodes staging axillary surgical procedure was evaluated in the study. Biopsies of sentinel lymph, with or without the complementary nodes, were classified as sentinel lymph node biopsy, otherwise it was described as an axillary lymph node dissection. Data on the type of surgical incision and skin resection were also collected. Intraoperative evaluation of resection margins with frozen sections was reported and evaluated in the study. Patients receiving intraoperative margin evaluation were considered as the standard group. Using the conventional surgical technique, all surgical specimens were sent for an intraoperative histopathological analysis that macroscopically evaluated the tissue. In addition, a microscopical study was performed based on the pathologist’s judgement. The number of further margins resected after intraoperative evaluation was also reported. When the surgeon removed, an extra layer of tissue from inside excision cavity is defined as the cavity shave group [13]. With the cavity shave technique, the lesion was removed close to the tumor. After the first resection, all margins were widened in order to clean the whole cavity from possible infiltrate [13]. Cases of intraoperative specimen radiography were reported from clinical notes and analyzed.

Awake surgery included all the procedures with the administration of local anesthetics or regional anesthesia, with or without mild sedation, and with spontaneous breathing. Data of the type of anesthesia and ASA score were reported from anesthetic records.

Tumor dimensions measured as the maximum diameter and expressed in millimeters from the final pathological examination report. Breast cancer prognostic and predictive factors included estrogen receptor (ER), progesterone receptor (PR), and Ki67 index, which were reported from pathological reports and expressed as percentages of positive cells. Overexpression of the Her2 gene (HER2+) was reported by pathological examination and identified by IHC or by FISH, as indicated by the recommendations of the 2018 ASCO/CAP. In addition, tumor staging was collected from histological reports, classified according the NCCN (National Comprehensive Cancer Network) guidelines [14]. Surgical margin (in millimeters) was defined as the distance between abnormal cells and the resection margins. A negative margin was considered “no ink on tumor” for invasive cancer and at least 2 mm for ductal carcinoma in situ. Re-operation for positive margins was reported from clinical notes within 120 days.

Surgical time (defined as time in operating room) was collected from operative records.

### Statistical Analysis

Data were recorded using an EXCEL (version 16.78, 2023) database (Microsoft, Redmond, Washington DC, USA). Statistical analysis was performed using the SPSS statistical package, version 23.0 (SPSS Inc., Chicago, IL, USA). Categorical variables were reported as the mean and standard deviation. The Mann–Whitney U-test was used to compare two different groups.

Variables presented as numbers and percentages were analyzed using the Student’s *T*-test for quantitative variables and the Chi-squared test (also known as Pearson’s test or Fisher’s exact test, depending on group size) for categorical dichotomous variables. For non-dichotomous variables, the Monte Carlo test were adopted. Variables with a *p*-value < 0.05 were considered statistically significant. The median value of surgical time was used as the cut-off to transform the continuous variables to dichotomous (considering inferior or not). The overall median time of operatory room occupancy was used to divide the population into two groups and perform multivariate analysis. A logistic regression statistical model, considering all the significative variables, was used to estimate the effect of factors on surgical time.

## 3. Results

From September 2021 to September 2023, 594 patients with mean age of 64.13 ± 13.63 years, underwent breast conserving surgery due to diagnosis of breast cancer and included in the study. The mean of weight and height were, respectively, 67.85 ± 13.65 kg and 161.62 ± 6.30 cm, and the BMI 25.98 ± 5.15 kg/m^2^.

In total, 180 (30.3%) underwent a cavity shave (CS-Group) and 414 (69.7%) received intraoperative evaluation of margins and considered as the control group (C-Group). Out of 180 cases of the CS-group, 100 (76.3%) had a diagnosis of ductal carcinoma, 25 (19.1%) lobular, and 6 (4.6%) other subtypes of breast carcinoma. In the C-group type of carcinoma were 277 (82.4%), 48 (14.3%), and 11 (3.3%), respectively, without a significant difference between groups (*p* = 0.304). Cases of ductal carcinoma in situ were significantly higher in the CS-Group with 32 patients (17.7%) versus 41 (9.9%) in the control group (*p* = 0.009). The number of multifocal lesions was significantly higher in the CS-Group 16 (8.8%) versus 18 (4.3%) in the C-group and the relative *p* was 0.029 (Table 1). All data regarding pre-operative diagnosis and predictive/prognostic factors are shown in Table 1.

The type of surgical incision chosen by surgeon did not show any statistically difference between the two groups (*p* = 0.363). The site of lesions according to breast quadrant did not show any statistically significant difference and the relative *p*-value was 0.124. Distributions are displayed in Table 1. The removal of skin was statistically significant (*p* = 0.002), with a higher incidence in the CS-group 43 (23.88%) vs. 157 (37.92%) in the control group. In the CS-Group, 52 (39.4%) patients required wire-guided lesion localization before surgery, versus 141 (41.0%) in the C-group and *p*-value was 0.751. In the CS-group, 27 (15.0%) patients had specimen radiographs versus 37 (8.9%) in the C-group. This difference was statistically significant and the relative *p*-value was 0.029.

Intraoperative evaluation of sentinel lymph nodes was performed in 44 (24.5%) patients in the CS-group and in 220 (53.2%) in the C-group and the *p*-value was <0.001. Cases of axillary lymph node dissection were 9 (5.0%) in the CS-group versus 37 (8.9%) (*p* = 0.131). Omission of sentinel biopsy, according to cancer subtype, was 64 (35.5%) in the CS-group versus 59 (14.3%) in the C-group and the relative *p*-value was <0.001. The Monte Carlo test comparing axillary procedure had a significant difference, with a *p*-value < 0.001. In the CS-Group, in 64 (35.5%) patients axillary surgery was omitted, in 98 (54.4%) sentinel lymph nodes dissection, and in 9 (5%) axillary lymph nodes dissection was performed. In the control group, the frequency of axillary procedures was 59 (14.3%), 312 (75.4%), and 37 (8.93%), respectively (Table 1).

Tumor dimension, T staging, grading, and breast cancer predictive and prognostic factors did not show any statistically significant differences (Table 2).

The ASA score was comparable between groups and the *p*-value was 0.504. A *p*-value of 0.847 was reported for incidence of awake procedure performed between groups.

Nine patents (5.0%) needed a second resection due to positive margins in the CS-group. Cases with positive margins were 23 (5.6%) in the control group (*p* = 0.846). In the cavity shave group, 164 (91.1%) patients had negative margins; no-ink on tumor for invasive cancer and more than 2 mm for in situ lesions. In the control group, 337 (81.5%) had negative margins (*p* = 0.003). The distance between resection margin was 8.45 ± 3.51 mm in the CS-group and 7.4 ± 4.24 mm in the C-group (*p* = 0.003). The distance between resection margin and tumor is displayed in Table 3. Sixteen (8.9%) patients had focally positive or closer margins while there were 77 (18.5%) in the control group (*p* = 0.003). No difference was reported in terms of surgical re-excision (*p* < 0.846) (5% vs. 5.5%).

The operative time for the two techniques was 80.7 ± 4.5 min for the cavity shave group, while in the standard procedures, it was 102.3 ± 4.7 min and the relative *p*-value was <0.001. In 4 cases (2.2%), patients with positive margins presented with microfocal satellite lesions in the CS-Group. Only 1 patient (0.2%) presented with a microfocal satellite lesion, and the relative *p* was 0.039.

Multivariate analysis with the following variables was undertaken, skin resection (Wald 0.878, *p* = 0.645, OR 1.011), axillary surgical procedure (omission, Sentinel or axillary lymph node dissection) (Wald 1.110, *p* = 0.292; OR 1.213; CI 0.526–8.463), specimen radiography (Wald 0.363, *p* = 0. 730; OR 1.133; CI 0.556–2.310), in situ tumor (Wald 0.561, *p* = 0. 534; OR 1.277; CI 0.645–1.518), multifocal lesions (Wald 0.463, *p* = 0. 571; OR 0.769; CI 0.310–1.098), intraoperative evaluation of sentinel lymph node (Wald 6.508, *p* = 0.020; OR 1.816; CI 1.826- CI 1.098–3.004), and cavity shave (Wald 22.89, *p* = 0. 001; OR 2.909 CI 1.878–4.504). Cavity shave was a predictive factor of shorter surgical times.

We evaluated the surgical time, excluding patients with diagnosis of DCIS due to the higher percentage of specimen radiographies. In the CS-group, the mean surgical time was 70.1 + 3.8 versus in the C-group 91.6 + 3.6 min and, from the *T*-test, the *p*-value was <0.001, (Table 4).

We evaluated surgical time excluding patients with diagnosis of DCIS and patients subjected to intra-operative evaluation of SNLB. In this analysis, considering 255 cases, the CS-group mean surgical time was 67.9 + 3.8 versus in the C-group 81.6 + 2.8 min and from the *T*-test, the *p*-value was 0.006. (Table 4)

## 4. Discussion

We conducted a retrospective analysis to compare differences and potential advantages between cavity shave margins and intraoperative pathological examination of resection margins. We found that excision of cavity shave margins reduced the surgical time and the rate of positive margins by nearly 10%.

Several studies have shown similar oncological advantages [13,14]. In 2015, in a randomized clinical trial comparing cavity shave versus no further resection of resection margins, they reported a reduction in positive margins by approximately 50% [14]. Different from this prospective analysis, in our study patients not subjected to cavity shave underwent macroscopic, and if necessary microscopic by frozen section, intra-operative evaluation of resection margins with further resection if needed. This difference could be associated with the lower reduction rate in positive margins with the cavity shave technique. Kobbermann et al., in a retrospective study found that before the introduction of the cavity shaving technique, re-operation for positive margins was 42% and reduced to 22% with the introduction of the cavity shave [15]. Marudanayagam et al. found that routine cavity shaving was associated with a lower rate of positive margins (6% vs. 12%) [16].

In our analyses, despite reporting a significant reduction in the incidence of positive margins, we did not find a significative increase in re-operation rate. This may be because the present study considered in situ lesions and had a different approach to analysis. Presently, consensus guidelines recommend a margin threshold of 2 mm in DCIS; however, the decision for re-operation for positive margins is a clinical judgment [17]. For example, in cases of focally positive margins, especially in elderly women, we prefer to avoid re-operation and perform high-dose radiation therapy, having similar local control of the disease [18,19,20].

Although some physicians have argued that routine cavity shaving in breast conserving surgery may not be needed, particularly if margins are excised due to macroscopic or histological findings, studies have shown a higher incidence of micro-focal satellite lesions in routine cavity shaves [21]. The current studies showed similar findings. Identifying potential multifocal breast cancer could be helpful for physicians to tailor treatments and follow-up. The need and rationale for re-operation for positive margins due to micro-satellite lesions was not the aim of this study.

Several studies have investigated surgical time [21,22]. In our study, we reported a significant reduction in surgical time of approximately 10 min with cavity shave versus intraoperative evaluation of surgical margins. Many factors could influence surgical time, such as intraoperative evaluation of sentinel lymph node, specimen radiography, and axillary surgical procedures. However, in multivariate analysis, the cavity shave method and intraoperative evaluation of the sentinel lymph node were found to be predictive of longer surgical time with an OR 2.9 and 1.8, respectively. In order to avoid potential bias, we excluded patients with intraoperative evaluation of the sentinel lymph node. We also excluded patients with DCIS lesions due to them having a significantly higher percentage of specimen radiography, and the cavity shave mean of surgical time was significative lower 67.9 + 33.8 versus 81.6 + 22.8 min for intraoperative evaluation of resection margins [22]. Mohamedahmed et al., in a previous analysis, reported a longer surgical time when cavity shaving was performed (79 ± 4 min vs. 67 ± 3 min, mean difference 12.14, *p* = 0.002) [23]. Monib et al. found that cavity shaves ensure microscopic clearance with no significant increase in operating time [24]. Despite this comparison of patients subjected to cavity shave or not, surgical times declared in these studies were comparable with time reported in our analysis. The control group in our analysis underwent intraoperative pathological examination of resected margins that despite a longer surgical time presented a lower incidence of positive margins compared to the aforementioned methods [24].

The cavity shave technique is preferred in patients with DCIS. In a randomized clinical trial, Howard-McNatt et al. reported that routine cavity shaves reduce positive margin rates in a group of patients [25]. Also, in previous single and multi-institutional trials, a similar result was reported [26]. DCIS lesions are known to have a different growth pattern and they are usually associated with micro-calcifications rather than nodular lesions [27]. The high percentage of specimen radiograms performed in the cavity shave group could be due to the increased rate of ductal carcinoma in situ and microcalcifications associated with these lesions. The lack of the nodule and the missed possibility of having tactile feedback may have led the surgeon to choose to excise all resected margins, typical of the cavity shaving technique. According to the current literature, DCIS are rarely associated with nodular lesions [28]; differently, invasive tumors manifest as nodular lesions in roughly 75% of cases [29].

Although most tumors are non-palpable, due to early diagnosis, they can often be felt as solid nodules in the intraoperative setting; this will guide the resection of the lesion and further margin excision.

Lack of tactile perception of the mass due to the absence of a proper nodule leads intraoperative uncertainty and removal of more tissue than planned; even with reliable pre-operative radiological findings and verified presence of microcalcifications and/or metallic clips by X-ray of the specimen, surgeons are more prone toward a wider resection to maximize the probability of an R0 excision.

Upon those observations, the cavity shave technique is now preferred in most DCIS. In the current literature, only one study proved the advantage of the cavity shave technique on a sample of 109 patients [30,31]. In this study, they reported a significant reduction in positive margins, but an analysis on the re-excision rate has not been performed. Margins < 2 mm are defined positive in in situ carcinoma, however, not all patients undergo second surgery for margin widening.

In our opinion, the “cavity shave” technique should be the preferred choice in nodular lesions; further studies are needed to evaluate whether cavity shave can lower re-operative rates in this subgroup.

We also believe that cavity shaving in association with specimen radiography is the best tool to reduce the incidence of positive margins in patients with DCIS.

Our results highlight how the cavity shave technique is associated with a significant reduction in the operative time, leading to a concomitant reduction in the costs and a better use of the available resources. The economic outcome was not quantified, being out of the scope of our study. Another advantage on the overall costs would be the reduction in the needed intraoperative histopathological examinations.

The studied technique most probably reduces the surgical time, also resulting in a shorter time of anesthesia, therefore, less operative and immunological stress [32,33].

Comparing the incidence of positive margins in our study with the current literature, we observed a significant reduction in the number of margins infiltrated by the neoplasia [15,16]. Based on those findings, either the cavity shave technique or the intraoperative histopathological examination of the specimen is necessary in order to reduce the rate of reintervention. Looking at the quality of life, it is clear how second surgery often has a negative impact on the psychophysical state of patients with a cancer diagnosis. Despite of the costs and the operative time, as surgeons, we should aim to minimize the risk of positive margins to reduce the need of re-excision; it is, nevertheless, equally important to avoid overtreatment and unnecessary tissue removal as this could cause reduced esthetic results and still have negative repercussions of the psychological state of patients. The main limitation of the study is the retrospective nature. In fact, as can be seen from the study, the choice of technique was entrusted to the surgeon’s preference based on the type of lesion and probably also on experience. Furthermore, as this study is retrospective, it meant that we did not have data on the aesthetic result.

## 5. Conclusions

The cavity shave strategy seems to be correlated with a lower incidence of positive margins, reducing surgical time when compared to intraoperative evaluation of resection margins in patients subjected to conserving breast surgery both for ductal carcinoma in sisu and invasive cancer. The cavity shave technique seems to be the strategy of choice for non-nodular lesions and in those specimen’s micro-calcification on radiological examination. Until a large prospective randomized clinical trial to confirm those advantages, it is the opinion that either cavity shave or intraoperative evaluation of resection margins must be performed to reduce the incidence of positive resection margins and re-excision.

## Figures and Tables

**Table 1:** Tumor pre-operative characteristics and intra-operative findings between groups.

	CS-Group *n* = 180	C-Group *n* = 414	*p*-Value
Multifocality	16 (8.88%)	18 (4.35%)	0.029
Multicentricity	0	5 (1.21%)	0.139
Wire-guided localization	52 (39.4%)	141 (41.0%)	0.751
Type of incision			0.363
Radial	92 (51.11%)	213 (51.45%)	
Periareolar	18 (10%)	70 (16.90%)	
Paraareolar	29 (16.11%)	49 (11.83%)	
Batwing	6 (3.33%)	7 (1.69%)	
Lesions quadrant			0.124
Upper outer quadrant UOQ	65 (36.11%)	165 (39.85%)	
UOQ-LOQ	20 (11.11%)	43 (10.38%)	
Upper inner quadrant UIQ	14 (7.77%)	55 (13.28%)	
LOQ-LIQ	14 (7.77%)	38 (9.18%)	
Lower outer quadrant LOQ	15 (8.33%)	35 (8.45%)	
Central Portion	15 (8.33%)	13 (3.14%)	
UOQ-UIQ	19 (10.55%)	43 (10.38%)	
Lower inner quadrant LIQ	5 (2.77%)	8 (1.93%)	
Specimen radiographs	27 (15.00%)	37 (8.94%)	0.029
Removal of skin	43 (23.88%)	157 (37.92%)	0.002
Intraoperative evaluation SNLB	44 (24.44%)	220 (53.14%)	<0.001
Axillary surgery			<0.001
SNLB	98 (54.44%)	312 (75.36%)	
ALND	9 (5.00%)	37 (8.93%)	
Omission	64 (35.55%)	59 (14.25%)	

SNLB: sentinel lymph node biopsy; ALND: Axillary lymph nodes dissection.

**Table 2:** Tumor staging, grading, and breast cancer prognostic and predictive factors between groups.

	CS-Group *n* = 180	C-Group *n* = 414	*p*-Value
Tumor diameter mm	15.37 ± 10.85	14.45 ± 8.24	0.349
ER %	65.91 ± 36.51	73.90 ± 31.68	0.016
PR %	45.17 ± 45.76	51.13 ± 38.81	0.122
Ki67%	19.11 ± 15.90	20.47 ± 16.61	0.416
HER2			0.093
Score 0	56 (31.11%)	174 (42.03%)	
Score 1	62 (34.44%)	133 (32.12%)	
Score 2	5 (2.77%)	34 (8.21%)	
Score 3	8 (4.44%)	24 (5.79%)	
Tumor grading			0.871
Grade 1	32 (17.77%)	75 (18.11%)	
Grade 2	51 (28.33%)	137 (33.09%)	
Grade 3	48 (26.66%)	123 (29.71%)	
T staging			0.139
Tis	13 (7.22%)	15 (3.62%)	
T1a	11 (6.11%)	27 (6.52%)	
T1b	32 (17.77%)	78 (18.84%)	
T1c	46 (25.55%)	160 (38.64%)	
T2	34 (18.88%)	96 (23.18%)	
T3	1 (0.55%)	1 (0.24%)	
T7	1 (0.55%)	2 (0.48%)	
N staging			0.079
N0	81 (45.00%)	253 (61.11%)	
N1a	13 (7.22%)	68 (16.42%)	
N1b	0 (0.0%)	0 (0.0%)	
N1c	0 (0.0%)	1 (0.24%)	
N2	2 (1.11%)	16 (3.86%)	
N3	1 (0.55%)	7 (1.69%)	

ER: Estrogen receptors; PR: Progesterone receptors; Ki67%: proliferation index; HER2: Human epidermal growth factor receptor.

**Table 3:** Margin evaluation between groups.

	CS-Group *n* = 180	C-Group *n* = 414	*p*-Value
Resection margin distance			
Deep margin mm	9.49 ± 2.17	9.04 ± 2.82	0.036
Superficial margin mm	9.79 ± 1.39	9.31 ± 2.50	0.004
Lateral margin mm	9.49 ± 2.16	9.32 ± 2.42	0.406
Medial margin mm	9.29 ± 2.52	9.35 ± 2.40	0.776
Upper margin mm	9.77 ± 1.49	9.24 ± 2.53	0.002
Lower margin mm	9.22 ± 2.58	9.27 ± 2.51	0.842
Closer margin			0.049
Negative	164 (91.1%)	337(81.5%)	
Deep margin	2 (1.11%)	22 (5.31%)	
Superficial margin	2 (1.11%)	10 (2.41%)	
Lateral margin	3 (1.66%)	15 (3.62%)	
Medial margin	3 (1.66%)	5 (1.21%)	
Upper margin	1 (0.55%)	12 (2.89%)	
Lower margin	5 (2.7%)	13 (3.14%)	
Multiple positive margins	13 (7.22%)	37 (8.93%)	0.430

**Table 4:** Operation time between groups.

	CS-Group	C-Group	*p*-Value
Overall	80.7 ± 4.5	102.3 ± 4.7	<0.001
Excluding DCIS	70.1+ 3.8	91.6 + 3.6	<0.001
Excluding DCIS and IO SNLB *	67.9 + 3.8	81.6 + 2.8	0.006

Ductal carcinoma in situ, SNLB: sentinel lymph node biopsy. * intra-operative evaluation of SNLB.

## Data Availability

The data presented in this study are available upon request from the corresponding author subject to valid justification.
